# Segmentation of the Proximal Femur from MR Images using Deep Convolutional Neural Networks

**DOI:** 10.1038/s41598-018-34817-6

**Published:** 2018-11-07

**Authors:** Cem M. Deniz, Siyuan Xiang, R. Spencer Hallyburton, Arakua Welbeck, James S. Babb, Stephen Honig, Kyunghyun Cho, Gregory Chang

**Affiliations:** 1 Department of Radiology, New York University School of Medicine, New York, NY 10016 USA; 2 Bernard and Irene Schwartz Center for Biomedical Imaging, New York University School of Medicine, New York, NY 10016 USA; 3 Center for Data Science, New York University, New York, NY 10012 USA; 4 Harvard College, Cambridge, MA 02138 USA; 5 Osteoporosis Center, Hospital for Joint Diseases, New York University Langone Medical Center, New York, NY 10003 USA; 6 Courant Institute of Mathematical Science, New York University, New York, NY 10012 USA

## Abstract

Magnetic resonance imaging (MRI) has been proposed as a complimentary method to measure bone quality and assess fracture risk. However, manual segmentation of MR images of bone is time-consuming, limiting the use of MRI measurements in the clinical practice. The purpose of this paper is to present an automatic proximal femur segmentation method that is based on deep convolutional neural networks (CNNs). This study had institutional review board approval and written informed consent was obtained from all subjects. A dataset of volumetric structural MR images of the proximal femur from 86 subjects were manually-segmented by an expert. We performed experiments by training two different CNN architectures with multiple number of initial feature maps, layers and dilation rates, and tested their segmentation performance against the gold standard of manual segmentations using four-fold cross-validation. Automatic segmentation of the proximal femur using CNNs achieved a high dice similarity score of 0.95 ± 0.02 with precision = 0.95 ± 0.02, and recall = 0.95 ± 0.03. The high segmentation accuracy provided by CNNs has the potential to help bring the use of structural MRI measurements of bone quality into clinical practice for management of osteoporosis.

## Introduction

Osteoporosis is a public health problem characterized by increased fracture risk secondary to low bone mass and microarchitectural deterioration of bone tissue. Hip fractures have the most serious consequences, requiring hospitalization and major surgery in almost all cases. Early diagnosis and treatment of osteoporosis play an important role in preventing osteoporotic fracture. Bone mass or bone mineral content is currently assessed most commonly via dual-energy x-ray absorptiometry (DXA)^1,2^. Over the years, cross-sectional imaging methods such as quantitative computed tomography (qCT)^3–9^ and magnetic resonance imaging (MRI)^10–14^ have been shown to provide useful additional clinical information beyond DXA secondary to their ability to image bone in 3-D and provide metrics of bone structure and quality^15^.

MRI has been successfully performed *in vivo* for structural imaging of trabecular bone architecture within the proximal femur^16–18^. MRI provides direct detection of trabecular architecture by taking advantage of the MR signal difference between bone marrow and trabecular bone tissue itself. Osteoporosis related fracture risk assessment using MR images requires image analysis methods to extract information from trabecular bone using structural markers, such as topology and orientation of trabecular networks^19–21^, or using finite element (FE) modeling^22–24^. Bone quality metrics derived from FE analysis of MR images are shown to correlate with high resolution qCT imaging, and may reveal different information about bone quality than that provided by DXA^18^. These technical developments overlay the significance of image analysis tools to determine osteoporosis related hip fracture risk.

Initial studies of MRI assessment of bone quality in proximal femur focused on quantification of parameters within specific regions of interest (ROI), such as the femoral neck, femoral head, and Ward’s triangle, for extracting fracture risk relevant parameters^18^. More recently, investigation of the whole proximal femur has been proposed as a way to assess the mechanical properties or strength of the whole proximal femur, rather than just a subregion^25–27^. The latter, however, requires manual segmentation of the whole proximal femur^18,28^ on MR images by an expert. Given the large number of slices for a single subject acquired by MRI during a scan session, time-consuming manual segmentation of proximal femur can hinder the practical use of MRI based hip fracture risk assessment. In addition, manual segmentation may be subject to inter-rater variability. Automatic segmentation of the whole proximal femur would help overcome these challenges.

In previous studies, hybrid image segmentation approaches including thresholding and 3D morphological operations^29^ as well as deformable models^30,31^ and statistical shape models^32^ have been used to segment the proximal femur from MR images. These approaches developed automated segmentation frameworks based on sophisticated algorithms. Deformable models achieved the mean accuracy of 1.44 ± 1.1 mm for the segmentation of the femur and hip bone from MR images^30^. Combining piecewise registration with deformable models resulted in sensitivities ~0.88 from clinical proximal femur MR images^31^. Moreover, statistical shape models achieved an average symmetric surface distance (ASD) of 1.21 ± 0.53 mm in the femur segmentations^32^. Even though these frameworks achieve reasonable femur segmentations from MR images, their use is limited by the time required to obtain proximal femur segmentations and by the robustness on a large variation of femur shapes.

The use of convolutional neural networks (CNNs) has revolutionized image recognition, speech recognition and natural language processing^33^. Deep CNNs have recently been successfully used in medical research for image segmentation and computer aided diagnosis^34^. In contrast to previous approaches of segmentation of proximal femur in MR images which rely on the development of hand-crafted features^29–31^, deep CNNs learn increasingly complex features from data automatically. The first applications of CNNs in medical image segmentation used pyramidal CNN architectures^34^ based on the information from local regions around a voxel as an input (patches) to predict whether the central voxel of the input patch belongs to a foreground or not. In a study using structural MRIs, Hallyburton *et al*. used pyramidal CNN architectures for segmenting the proximal femur to achieve moderate segmentation results with dice similarity coefficient (DSC) ~0.70^35^. These approaches are limited by the size of the receptive field of the networks and by the time required for CNN training and inference, especially for volumetric datasets.

Developments in image segmentation using fully convolutional network architectures have emerged resulting in more accurate pixel-wise segmentations^36–38^. These networks used encoder-decoder type architectures, where the role of the decoder network is to project the low resolution encoder feature maps to high resolution feature maps for pixel-wise classification. Encoder-decoder based CNN architectures have been recently used extensively in the biomedical field providing accurate image segmentation^34^. The use of network architectures for segmenting 3D musculoskeletal images have been focused on developing learning-based segmentation models in 2D and using post-processing to capture 3D tissue information for generating 3D segmentation mask. For example, 2D encoder-decoder network architectures were accompanied by 3D connected component analysis^39^ or 3D simplex deformable modeling^40^ to provide a final 3D segmentation mask. Moreover, cascading 2D CNN with intermediate statistical shape modeling for generating a smaller patch-based inputs for a 3D CNN model has been proposed^41^ for segmenting the knee menisci. However, it is not clear if the need for combining 2D CNN outputs with image segmentation approaches arises from inherent selection of 2D CNN as a segmentation method or not. In addition, incorporation of modeling approaches in segmentation pipeline could impede with the benefits of end-to-end learning-based segmentation approaches. Given that the CNN are capable of modeling nonlinear interactions between the musculoskeletal MR image and the segmentation mask, 3D interactions required for accurate tissue segmentation and provided by the combination of 3D image processing methods in the previous studies^39–41^ might in the future be captured effectively by end-to-end 3D CNN segmentation model. This study lays the groundwork for such potential future investigation.

In this work, we propose to investigate CNN architectures based on the U-net^38^ and the 3D extension of the U-net^42^, and compare their performance for automatic segmentation of the proximal femur on MR images against the reference standard of expert manual segmentation. Different U-net based CNN architectures were implemented by changing the number of feature maps and encoding-decoding layers to analyze the effect of architecture design parameters on proximal femur segmentation performance. In addition, we extended the CNN architectures by concatenating dilated convolutions^43,44^ with different dilation rates in the center layer of the encoder-decoder architecture.

## Results

### Comparison of CNN Performance

**Figure 1:**
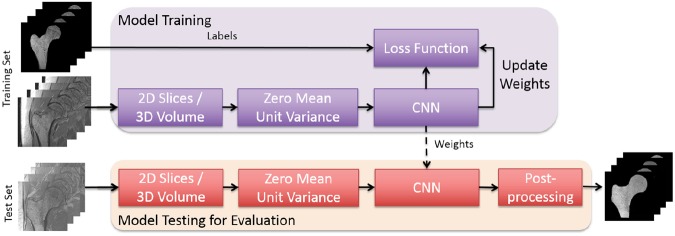
Overview of the proposed learning algorithm for an automatic segmentation of the proximal femur. Training CNN yields automatic proximal segmentation model that is used in model evaluation on a test dataset. The output of the model is the probability of the bone which is used to obtain the proximal femur segmentation mask using a threshold.

**Figure 2:**
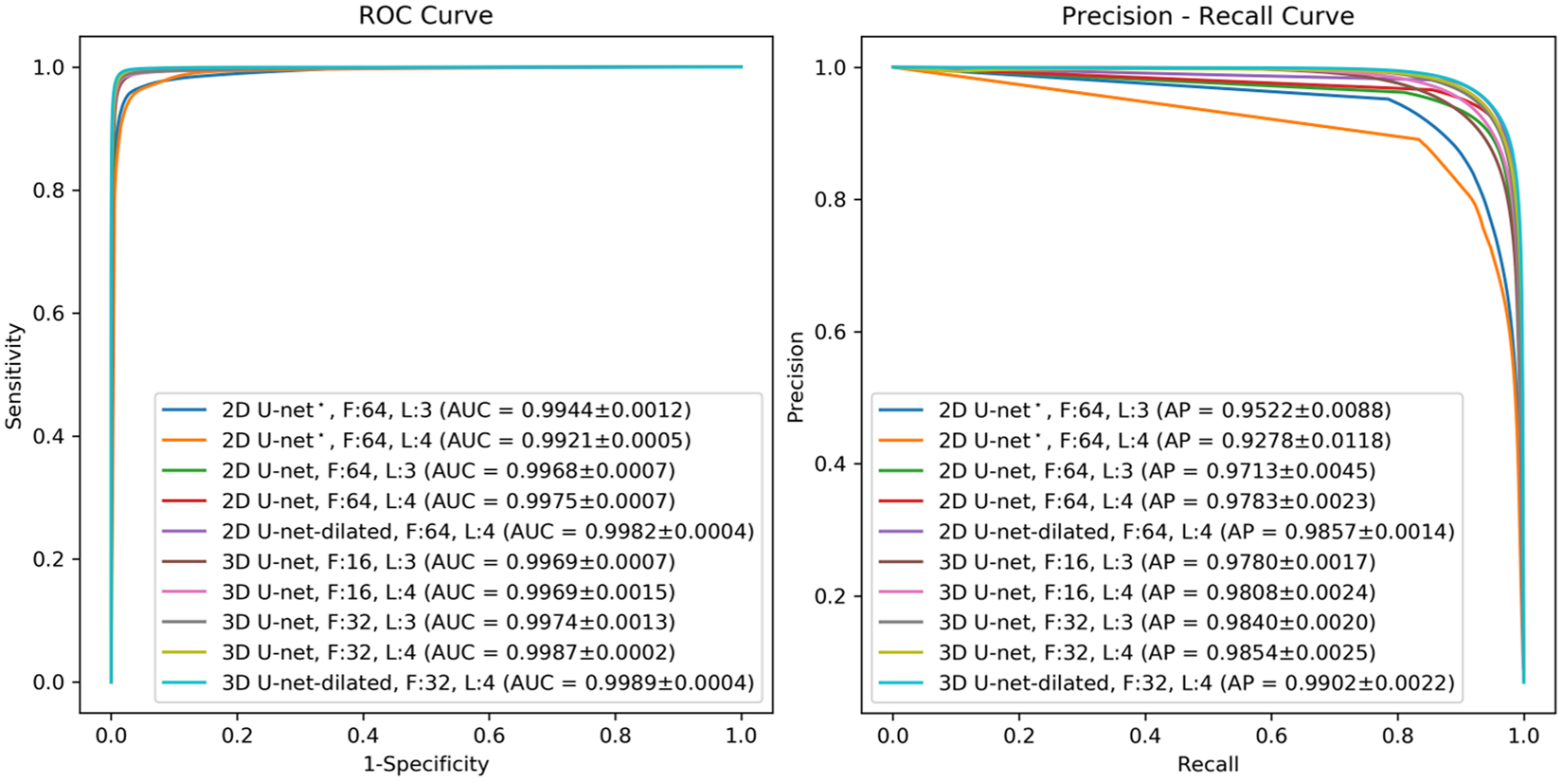
ROC and Precision-Recall Curve for 2D and 3D CNN segmentation models. Left panel shows the receiver operating characteristics (ROC) curves of different CNNs modeled in this work. The number of initial feature maps (F) and layers (L) in the contracting/expanding paths are presented in the legend with the area under the curve (AUC). Right panel shows the precision- recall curves of modeled CNNs. In the legend, cross-validation average precision (AP) is presented for comparison of different models. * indicates the 2D CNN with unpadded convolutions.

Various CNN architectures have been used for automatic segmentation of biomedical images^34^. In this study, two supervised deep CNN architectures based on 2D convolution (2D CNN) and 3D convolution (3D CNN) were used and evaluated for automatic proximal femur segmentation on MR images. The best performing CNN architecture for both 2D CNN and 3D CNN was improved by concatenating dilated convolutions with different dilation rates to study the effect of architecture changes in segmentation performance. An overview of the proposed approach for automatic segmentation of the proximal femur is presented in Fig. 1. Receiver operating characteristics (ROC) and precision-recall curve (PRC) analysis of modeled CNNs on the dataset are presented in Fig. 2 using the mean curves from 4-fold cross-validation. We use the area under the PRC (AP: average precision) as a measure of classifier’s performance for comparing different CNNs. The 3D CNN-dilated with 32 initial feature maps and 4 layers each in the contracting/expanding paths and concatenation of feature maps obtained with dilation rates *r* = 1, 2, 4, 8 outperformed the other CNNs with area under the ROC curve (AUC) = 0.999 ± 0.0 and AP = 0.990 ± 0.002. This model achieved the highest accuracy on the segmentation of the proximal femur without post-processing, and it exceeded the performance of 2D CNNs which achieved AUC = 0.998 ± 0.001 and AP = 0.978 ± 0.002. The performance of the CNN model improved as the number of layers and feature maps increased for both 2D and 3D CNN.

PRC analysis provides a means of evaluating the performance of automatic segmentation algorithms and selecting a suitable decision threshold. The output of a CNN defines the probability of a voxel belonging within the proximal femur. Using PRC analysis, the optimal threshold is selected for each CNN to distinguish proximal femur bone voxels from background when comparing the performance of CNNs. The optimal operating point for each CNN was selected by choosing the point on the PRC that has the smallest Euclidean distance to the maximum precision and recall. There are other ways of choosing the optimal operating point such as using the Youden index^45^ or defining the smallest Euclidean distance to the maximum sensitivity and specificity via performing ROC curve analysis. We chose to use the smallest Euclidean distance to the maximum precision and recall to prevent under-segmentation (when the recall is low) and over-segmentation (when the precision is low). The voxels having higher probabilities then selected threshold is predicted as belonging within the proximal femur and the rest as background. The optimal threshold was applied to the segmentation probability maps to calculate a binary segmentation mask. The binary segmentation map from each individual is used to compare the CNN models by analyzing performance metrics. In the 2D CNN, additional post-processing was applied to the segmentation mask since CNN results included misclassified bone regions. From the models without post-processing, the 3D CNN-dilated with 32 initial feature maps, 4 layers and dilation rates *r* = 1, 2, 4, 8 resulted in the highest DSC = 0.953 ± 0.016 with precision = 0.954 ± 0.017, and recall = 0.953 ± 0.030. This CNN achieved the lowest average symmetric surface distance (ASD) = 0.39 ± 0.19 mm with the maximum surface distance (MSD) = 7.88 ± 4.33 mm (Table 1).

**Table 1:** Segmentation results of different network architectures for the segmentation of proximal femur.

Network	DSC ↑	Precision ↑	Recall ↑	ASD [mm] ↓	MSD [mm] ↓
2D CNN*, F:64, L:3	0.886 ± 0.055	0.890 ± 0.080	0.889 ± 0.056	6.15 ± 3.61	65.78 ± 5.78
2D CNN*, F:64, L:4	0.864 ± 0.044	0.872 ± 0.061	0.860 ± 0.060	6.82 ± 3.00	64.89 ± 6.36
2D CNN, F:64, L:3	0.924 ± 0.032	0.920 ± 0.041	0.930 ± 0.045	3.13 ± 1.76	54.40 ± 6.72
2D CNN, F:64, L:4	0.937 ± 0.026	0.932 ± 0.037	0.943 ± 0.036	2.13 ± 1.23	42.22 ± 5.52
2D CNN-dilated ^†^, F:64, L:4	0.946 ± 0.022	0.948 ± 0.024	0.944 ± 0.034	1.75 ± 1.24	40.03 ± 8.37
3D CNN, F:16, L:3	0.927 ± 0.032	0.931 ± 0.029	0.927 ± 0.063	0.66 ± 0.32	10.62 ± 6.85
3D CNN, F:16, L:4	0.935 ± 0.028	0.938 ± 0.026	0.936 ± 0.053	0.59 ± 0.39	9.75 ± 6.56
3D CNN, F:32, L:3	0.942 ± 0.026	0.944 ± 0.022	0.942 ± 0.052	0.50 ± 0.25	11.97 ± 7.57
3D CNN, F:32, L:4	0.945 ± 0.029	0.948 ± 0.023	0.944 ± 0.052	0.45 ± 0.25	13.44 ± 13.14
3D CNN-dilated^†^, F:32, L:4	**0.953** ± **0.016**	**0.954** ± **0.017**	**0.953** ± **0.030**	**0.39** ± **0.20**	**7.88** ± **4.33**

Performance metrics are presented using the mean and the standard deviation that are calculated from individual subject segmentations. F is the number of initial feature maps, L is the number of layers. * indicates the 2D CNN with unpadded convolutions. ^†^ indicates the best performing CNN-dilated model that is presented here for concatenation of feature maps with dilation rates = 1, 2, 4, 8. The analysis of different CNN-dilated models can be found in Table 3.

**Figure 3:**
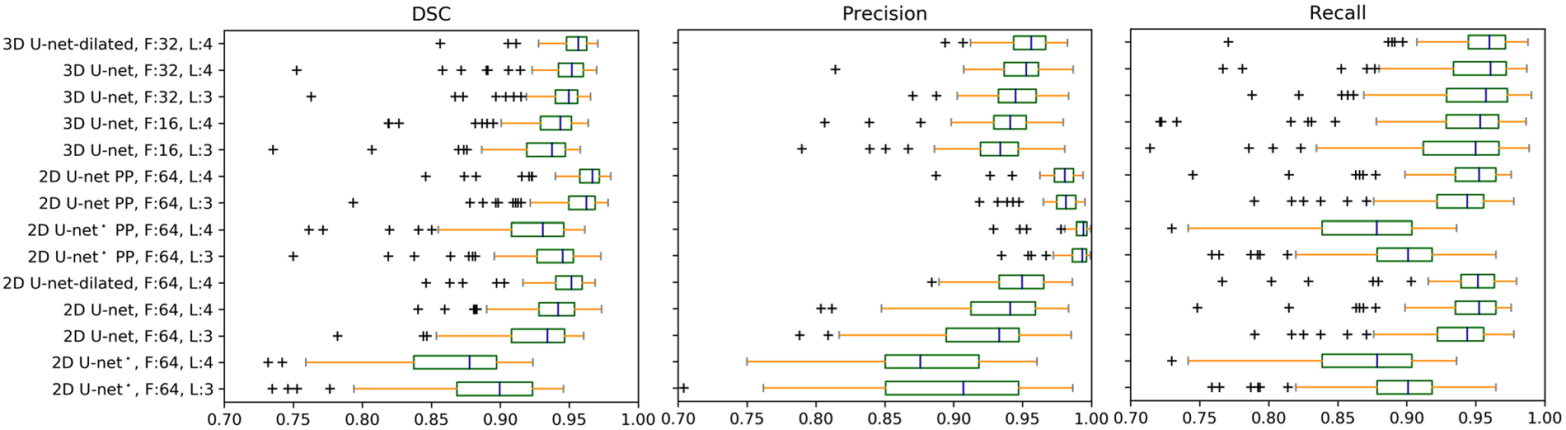
Box plots for dice score, precision and recall that are obtained from the binary segmentation map from each individual. F is the number of initial feature maps, L is the number of layers, PP is the post-processing. * indicates the 2D CNN with unpadded convolutions.

Analysis of performance metrics on individual subjects is illustrated in Fig. 3 and Table 1. Applying post-processing on the 2D CNN segmentation results improved the overall accuracy of the segmentation masks as indicated by the increase in DSC on average by 7% and by the decrease in ASD on average by 86% (Table 2). As indicated by Fig. 3, post-processing improves the precision on 2D CNNs; however, average recall was not affected by the post-processing significantly. In terms of ASD and MSD, the improved precision brings the 2D CNN with post-processing closer to the 3D CNN. The best performing 2D CNN with post-processing exceeds the precision and DSC of the best performing 3D CNN.

**Table 2:** The effect of post-processing (PP) in 2D CNN segmentation results.

Network	DSC ↑	Precision ↑	Recall ↑	ASD [mm] ↓	MSD [mm] ↓
2D CNN* PP, F:64, L:4	0.920 ± 0.040	**0.991** ± **0.010**	0.861 ± 0.060	0.72 ± 0.38	11.70 ± 3.74
2D CNN* PP, F:64, L:3	0.935 ± 0.034	0.990 ± 0.010	0.889 ± 0.056	0.62 ± 0.36	10.50 ± 3.23
2D CNN PP, F:64, L:4	**0.960** ± **0.022**	0.978 ± 0.015	**0.943** ± **0.036**	**0.39** ± **0.44**	**8.18** ± **5.87**
2D CNN PP, F:64, L:3	0.953 ± 0.027	0.979 ± 0.013	0.930 ± 0.046	0.47 ± 0.37	9.61 ± 4.60
2D CNN^†^, F:64, L:4	0.937 ± 0.026	0.932 ± 0.037	**0.943** ± **0.036**	2.13 ± 1.23	42.22 ± 5.52

Segmentation results of different network architectures are presented here. F is the number of initial feature maps, L is the number of layers. * indicates the 2D CNN with unpadded convolutions. ^†^ indicates the best performing 2D CNN model prior to post-processing from Table 1.

The analysis of the effect of dilation rate in segmentation accuracy is provided in Table 3 for the best performing 2D CNN and 3D CNN selected based on the cross-validation AP values presented in Fig. 2. The statistical analysis was performed on the AP values that are derived for the individual subject segmentations using the CNN model in which the subject was in the validation set during model training. Incorporating at least one dilated convolution with dilation rate larger than 2 resulted in significantly higher AP than the original CNN implementation (*p* < 0.05 for 2D CNN and *p* < 0.001 for 3D CNN, Table 4). This resulted increased segmentation accuracy with respect to the original CNN implementation. Moreover, the most benefit was obtained using a concatenation of feature maps with different dilation rates. Additionally, 3D CNN model (F:32, L:4 with dilated convolution) showed significantly higher AP than any 2D CNN method (F:64, L:4, with/without dilated convolution) (*p* < 0.01, Table 4).

**Table 3:** Segmentation results of different CNN architectures with dilated convolution for the segmentation of proximal femur.

Network	Dilation Rate (*r*)	AP ↑	DSC ↑	Precision ↑	Recall ↑	ASD [mm] ↓	MSD [mm] ↓
3D CNN F:32, L:4	1^†^	0.986 (0.002)	0.945 ± 0.029	0.948 ± 0.023	0.944 ± 0.052	0.45 ± 0.25	13.44 ± 13.14
	1, 2	0.988 (0.001)	0.950 ± 0.020	0.951 ± 0.017	0.949 ± 0.039	0.43 ± 0.23	8.77 ± 6.32
	1, 4	0.988 (0.002)	0.949 ± 0.019	0.948 ± 0.022	0.950 ± 0.036	0.43 ± 0.21	8.10 ± 4.73
	1, 8	0.988 (0.003)	0.948 ± 0.015	0.949 ± 0.023	0.948 ± 0.031	0.44 ± 0.19	8.05 ± 4.25
	1, 2, 4	0.988 (0.002)	0.948 ± 0.023	0.948 ± 0.021	0.949 ± 0.039	0.43 ± 0.24	8.14 ± 5.68
	1, 2, 4, 8	**0.992 (0.002)**	**0.953** ± **0.016**	**0.954** ± **0.017**	**0.953** ± **0.030**	**0.39** ± **0.19**	**7.88** ± **4.33**
2D CNN F:64, L:4	1^†^	0.979 (0.003)	0.937 ± 0.026	0.932 ± 0.036	0.943 ± 0.036	2.13 ± 1.22	42.22 ± 5.49
	1, 2	0.978 (0.002)	0.939 ± 0.025	0.937 ± 0.034	0.944 ± 0.037	2.04 ± 1.33	40.10 ± 7.34
	1, 4	0.984 (0.000)	0.943 ± 0.022	0.944 ± 0.026	0.944 ± 0.035	1.85 ± 1.13	39.70 ± 7.38
	1, 8	0.984 (0.002)	0.941 ± 0.023	0.941 ± 0.030	0.943 ± 0.034	2.04 ± 1.31	42.41 ± 7.60
	1, 2, 4	0.984 (0.005)	0.941 ± 0.025	0.941 ± 0.033	0.942 ± 0.034	2.02 ± 1.39	40.27 ± 7.76
	1, 2, 4, 8	0.986 (0.002)	0.946 ± 0.022	0.948 ± 0.024	0.944 ± 0.034	1.75 ± 1.24	40.03 ± 8.37

The best performing 2D CNN and 3D CNN models (based on cross-validation AP on Fig. 3) were used as a baseline for our experiments. AP values are derived for the individual segmentations and performance metrics are calculated from individual subject segmentations. F is the number of initial feature maps, L is the number of layers. ^†^ indicates the original CNNs presented in Table 1. Statistical analysis for comparing models using AP is presented in Table 4. AP data is presented using the median and interquartile range in parentheses.

**Table 4:** Statistical analysis results of comparing the AP difference between CNN models with different dilation rates. The segmentation results of these models are presented in Table 3.

	Dilation Rate (r)	2D CNN, F:32, L:4						3D CNN, F:64, L:4					
		1^†^	1, 2	1, 4	1, 8	1, 2, 4	1, 2, 4, 8	1^†^	1, 2	1, 4	1, 8	1, 2, 4	1, 2, 4, 8
2D CNN F: 32, L:4	1^†^		ns	***	**	*	***	***	***	***	***	***	***
	1, 2			ns	ns	ns	**	**	***	***	***	***	***
	1, 4				ns	ns	ns	ns	***	***	***	***	***
	1, 8					ns	*	ns	***	***	***	***	***
	1, 2, 4						ns	ns	***	***	***	***	***
	1, 2, 4, 8							ns	**	***	**	**	***
3D CNN F:32, L:4	1^†^								***	***	*	**	**
	1, 2									ns	ns	ns	ns
	1, 4										ns	ns	ns
	1, 8											ns	**
	1, 2, 4												*
	1, 2, 4, 8												

To assess the significant differences between CNN models, we used the paired-sample Wilcoxon signed-rank test with Holm correction for multiple comparisons. *p*-values are indicated in the table using the following convention: ns = *p*-value ≥ 0.05, *p-value < 0.05, **p-value < 0.01, and ***p-value < 0.001.

### Segmentation accuracy

**Figure 4:**
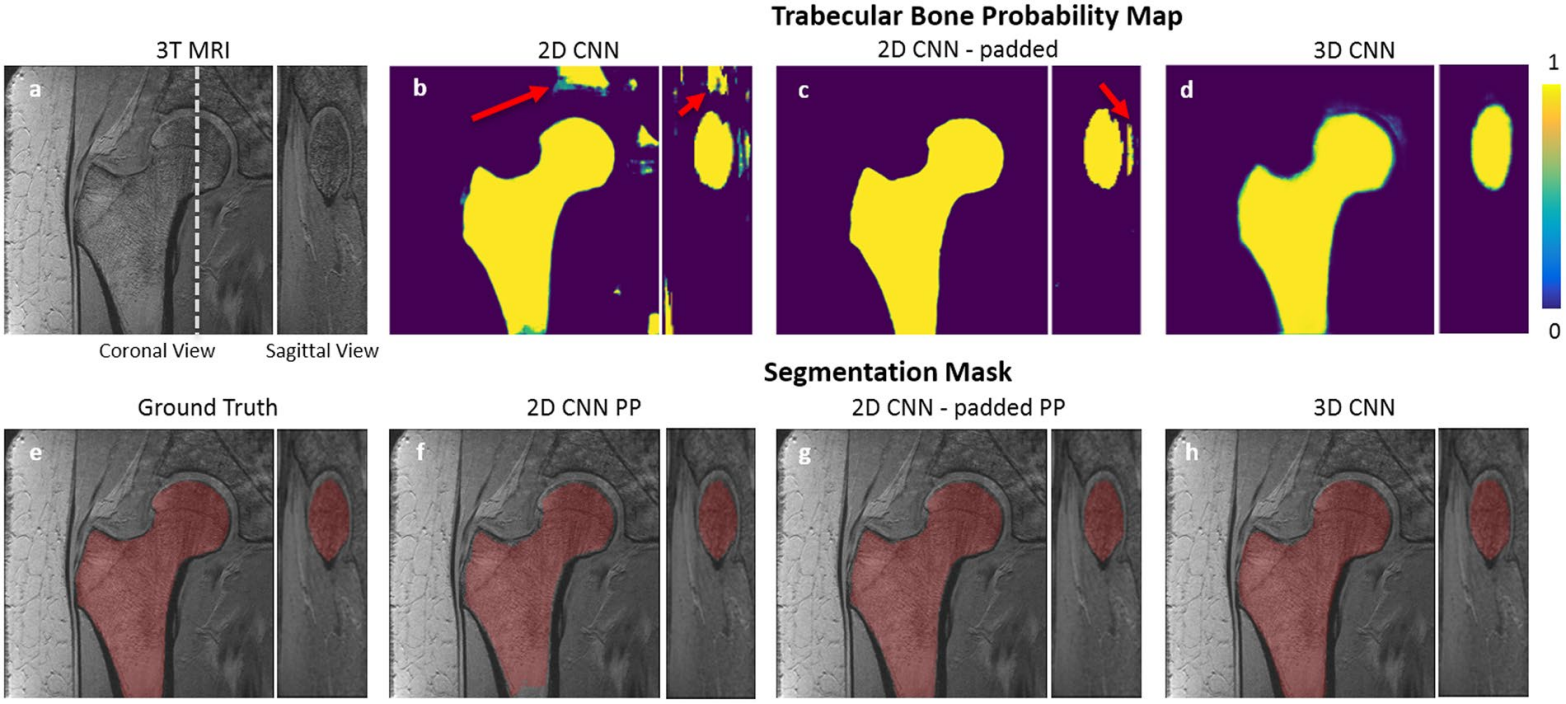
An example of the results using 2D CNN and 3D CNN. 3T MRI of the proximal femur (**a**) is shown with the ground truth/hand segmentation mask (**e**). The white dashed line represents the location where the sagittal view is displayed from the coronal view. The probability map produced by 2D CNN is presented in (**b**) and corresponding segmentation mask after post-processing is presented in (**f)**. Red arrow in (**b**) indicates a location which was misclassified by the 2D CNN. Using padded convolution provided superior segmentation (b vs c). Some of the misclassified regions in (**b**) are removed by using the padded convolution; however, there are still regions that are misclassified as indicated by the red arrow in (**c**). Misclassified regions were removed by post-processing using proximal femur connectivity and size prior information (**f** and **g**). Probability map produced by 3D CNN is presented in (**d**) and corresponding segmentation mask obtained by thresholding without post-processing is presented in (**h**).

Segmentation results on one of the subjects is shown in Fig. 4. The proximal femur bone probability map from the 2D CNN includes misclassified regions which are not part of the proximal femur (as indicated by the red arrow). Removing the small clusters of misclassified bone regions with post-processing clearly improved the segmentation accuracy and resulted in a well-connected 3D proximal femur (Fig. 4e). However, there are still misclassified locations remain, e.g. the bottom part of the proximal femur. In contrast to the 2D CNN, the 3D CNN automatically captures the global connectivity of the proximal femur during CNN training. This results in better delineation of the proximal femur on the trabecular bone probability map (Fig. 4c) which provides a segmentation mask resembling the ground truth with higher accuracy. Because of this, as opposed to the 2D CNN, additional post-processing step was not performed on the 3D CNN segmentation results.

**Figure 5:**
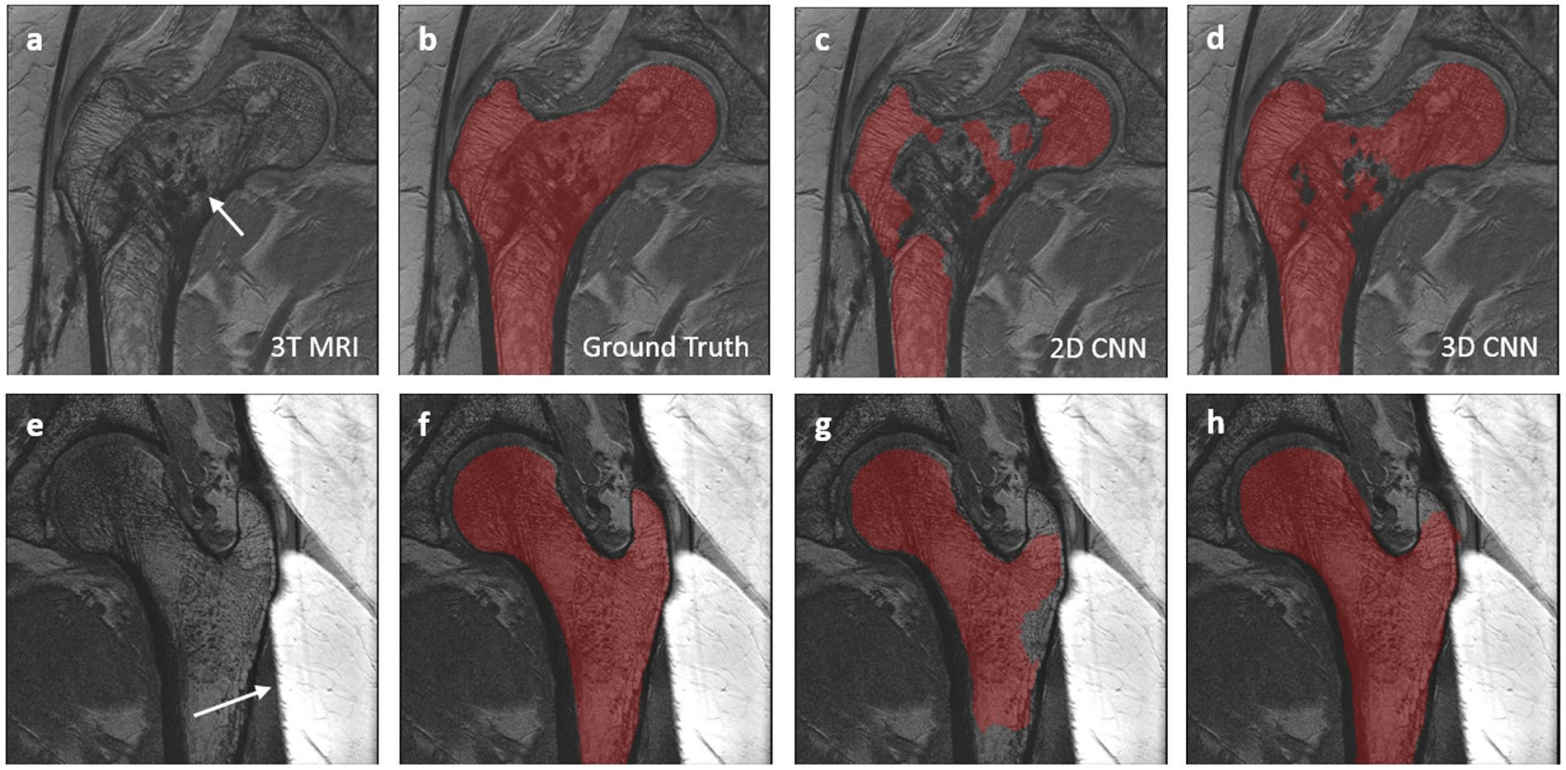
Examples of the suboptimal segmentation results. First row images are from a subject who has a bone cysts in the proximal femur. 3T MRI of the proximal femur in (**a**) is shown with the ground truth/hand segmentation mask overlaid in (**b**). Both 2D (**c**) and 3D (**d**) CNN were not capable of segmenting the proximal femur of this subject with high accuracy. Second row images are from an acquisition where there is a foldover artifact (indicated by the white arrow) that is not affecting the hand segmentation (**f**). However, foldover artifacts are affecting the accuracy of automatic proximal femur segmentations of both 2D and 3D CNN (**g**,**h**). These segmentation results remained suboptimal with minor improvements when dilated convolutions are used.

Examples of suboptimal proximal femur segmentation results are shown in Fig. 5. The MR image and segmentation maps provided in the first row are from a subject who had a bone cyst in the proximal femur and suffered from a hip fracture in early ages. Some of the hypointense foci (as indicated by the white arrow in Fig. 5a) are related to calcium deposition from the healing process. Compared to ground truth proximal femur segmentation (Fig. 5b), both 2D (Fig. 5c) and 3D (Fig. 5d) CNN were influenced by the hypointense regions within the proximal femur resulting inaccurate segmentation. The suboptimal segmentation results could be attributed to the low frequency of such subjects in our current dataset (1 in 86 subjects). In the second row of the Fig. 5, MR image acquisition is compromised by the fold-over artifacts (as indicated by the white arrow in Fig. 5e). Our dataset contains only 3 MR images with fold-over artifacts. These artifacts do not affect the hand segmentation and analysis of the proximal femur microarchitecture per se; however, learning-based automatic segmentation models are affected negatively, resulting in a subcomplete segmentation mask covering only the parts of the proximal femur. These segmentation mask errors are more pronounced on the 2D CNN results. Since CNN approaches learn and generalize from data, incorporating a range of subjects with different problems on the proximal femur and possible fold-over artifacts will enable more general CNN-based proximal femur segmentation models. In addition, combining the current loss function (Eq. 1) with a surface-based weighting, a variant of weight-map for the borders in ref.^38^, could improve the accuracy of segmentation as the surface of the proximal femur for both acquisitions resembles the general population experimented in this paper.

### Computational efficiency

Training each epoch takes approximately 5 minutes, 7 minutes and 7 minutes for the 2D CNN with unpadded convolution, 2D CNN and 3D CNN (for networks with 32 feature maps and 4 layers), respectively. The total time required for inference for the segmentation of data from one subject with central 48 coronal slices (covering the proximal femur) was approximately 18 seconds, 4 seconds and 5 seconds for 2D CNN with unpadded convolution, 2D CNN and 3D CNN (for networks with 32 feature maps and 4 layers), respectively. Dilated CNN models had a negligible increase in training and inference time compared to the original CNN. The increase in the inference time on the 2D CNN with unpadded convolution was due to the use of multiple patches (9 patches per 2D slice) for calculating the segmentation mask on the full field of view.

## Discussion

We present a deep CNN for automatic proximal femur segmentation from structural MR images. The automatic segmentation results indicate that the requirement of expert knowledge on location specifications and training/time for segmentation of the proximal femur may be avoided using CNNs. A Deep CNN for automatic segmentation can help bringing the use of proximal femur MRI measurements closer to clinical practice, given that manual segmentation of hip MR images can require approximately 1.5–2 hours of effort for high resolution volumetric datasets.

CNN-based automatic segmentation of MR images has been performed in the brain^46^, including for brain tumors^47^, microbleeds^48^, and skull stripping for brain extraction^49^. CNN-based automatic segmentation has also been used for the pancreas^50^ and for knee^40,51^. In recent years, automated segmentation of the proximal femur from MR images using a CNN begin to emerge in workshops^52^ and conferences^35^. Our results confirm previous results and further contribute to the field in two ways: (i) by examining data from an increased number of subjects, and (ii) by analyzing architectures that use 2D or 3D convolution in the concept of automated segmentation of the proximal femur from MR images. In the future, we expect the number of imaging applications of CNNs to rapidly increase, especially given the publicly available software libraries such as Tensorflow^53^ to create CNNs and the ability to execute the algorithm on commercially available desktop computers.

In our implementation of the segmentation algorithms, the use of 2D convolutional kernels could be one of the reasons for misclassified bone regions in 2D CNN and in its variant 2D CNN with unpadded convolution. Even though information from consecutive slices are incorporated in 2D CNN model training, global connectivity of the proximal femur may not be modeled properly using 2D convolution alone. Although we used post-processing to prevent misclassified small regions in 2D CNNs, the approach using 3D convolutional kernels (3D CNN) resulted in a better segmentation masks by directly modeling the 3D connectivity of the proximal femur during training. Avoiding the post-processing step in an automatic segmentation algorithm is crucial especially for segmentation tasks that aim to identify multiple regions. CNNs with 3D convolutional networks are computationally more demanding and can result in higher overfitting due to the increased number of weights to train. In all of our experiments, we used the validation error as an early stopping criterion to successfully overcome potential overfitting.

In addition to comparing the performance of segmentation models using 2D/3D convolution and different number of feature maps and layers, we performed experiments using dilated convolutions to increase the receptive field of the encoding path of the CNN. Our design choice of using dilated convolutions in the last layer of the encoding network was to increase the receptive field of the CNN to cover the whole image in a way that a possible missing global segmentation information can be captured effectively. It is expected that the global connectivity information in addition to the local information like texture is important for bone segmentation. We proposed to achieve this with minor changes to the original architecture so that the effect of dilated convolutions can be analyzed systematically. Incorporating dilated convolutions in the center of the CNN resulted in improved segmentations by gathering multi-scale global proximal connectivity information effectively. Dilated convolutions can also be used in multiple layers within the architecture in order to increase the receptive field and reduce the number of parameters.

In the 2D CNN with unpadded convolution, similar to the original U-net paper^38^, mirrored images were used during inference for calculating the probability of each voxel being part of the proximal femur. This resulted in inferencing on multiple patches covering the image and averaging the probability to calculate the output segmentation mask. We used multiple patches covering the image during inference only. Multiple patches from the mirrored images could also be used during training. This change in the training will result in increased training time as the number of training samples from each image per epoch will increase. Similarly, mirrored images can also be used during training, which removes the necessity of multiple calculations for averaging during inference. However, the increase in the input size of the network can result in an increased training time and a higher GPU memory requirement. On the other hand, using mirrored images for modeling will reduce the time required by inference and post-processing for 2D CNNs with unpadded convolution. We also implemented 2D CNNs with padded convolutions instead of unpadded ones, as done in 3D CNN. This modification was used to obtain segmentation outputs that have the same size as the input images. This removed the necessity of extracting multiple patches for calculating multiple segmentation probability maps and averaging them during inference.

This feasibility study lays the ground work for future studies which may involve patients who have diseases such as hip dysplasia, osteoarthritis, or femoroacetabular impingement, which all result in abnormal proximal femur morphology and whose clinical management can be influenced by quantitative measurements (e.g. center edge angle in hip dysplasia or alpha angle in femoroacetabular impingement). Automatic segmentation methods for the proximal femur in the future could be used to automate such measurements or help develop novel quantitative metrics of bone health. We are currently pursuing such projects at our medical center.

We note the existence of many other deep-learning based methods for automatic segmentation of MR images^34^. Recent work in the brain and spine have shown that automatic segmentation of brain subregions and lesions^54,55^ and intervertebral discs^56^ is possible. Specifically, Kamnitsas *et al*.^54^ applied a 3D CNN combined with a full connected conditional random field (CRF) (as post-processing to remove false positives) to successfully automatically segment MR images of brain lesions in subjects with traumatic brain injury, brain tumors, and ischemic stroke. Chen *et al*.^55^ used a novel voxelwise residual network built with 25 layers to automatically segment the hippocampus on brain MR images. Finally, Li *et al*.^56^ applied a fully convolutional network (FCN) with random modality dropout learning to automatically segment intervertebral discs on MR images. While comparison of our method with these other methods is beyond the scope of this work, as it would also require the proper implementation of the methods, the existence of multiple deep learning methods and also other automatic segmentation methods (atlas-based registration, machine learning-based methods for specific features) suggests that methodology comparison will be an important area of study in the future. Standards for study design and public datasets will have to be defined so that comparisons are fair and objective. The best automatic segmentation methodology may actually differ depending on the target tissue of interest as well as the imaging modality.

This study has limitations. First, even though we implemented multiple CNNs with different number of feature maps and layers, the automatic advanced hyperparameter optimization^57^ for the CNN training parameters was not implemented in the current study. In the future, the optimization of learning rate and the number of initial feature maps will be performed. We expect the misclassified proximal femur bone regions in 2D CNN will be mitigated; and in every network architectures this optimization will provide superior segmentation results. Second, image segmentation is a fast growing field with new architectures and approaches presented each year. We limited CNN architectures demonstrated in this work to cover current fundamental architectures^38,42^, in which their variants have been used extensively for biomedical image segmentation. Comparing our results with the recent architectural developments^46,58–60^ and using different loss functions^58,61,62^ instead of weighted cross-entropy is beyond the scope of this work. In the future, it will be important, not just for this work, but for the field of machine learning in general to compare CNN methods to the state-of-the-art non-CNN methods for automatic segmentation and image analysis. To the best of our knowledge, there is currently no publicly available non-CNN method for automatic segmentation of MR images of the proximal femur. It may be that a combination of CNN and non-CNN methods could provide the best performance for automatic image segmentation or analysis.

In conclusion, we compared two major CNN architectures that are being increasingly used for biomedical image segmentation for automatic segmentation of the proximal femur. For both architectures, we experimented the use of dilated convolutions in the center layer. Our experiments demonstrated the improved performance obtained using 3D and dilated convolutions, and post-processing in 2D CNN for automatic segmentation of the proximal femur. The automatic segmentation using CNNs has the potential to bring the use of structural MRI measurements into the clinical practice.

## Methods

### Convolutional neural networks

**Figure 6:**
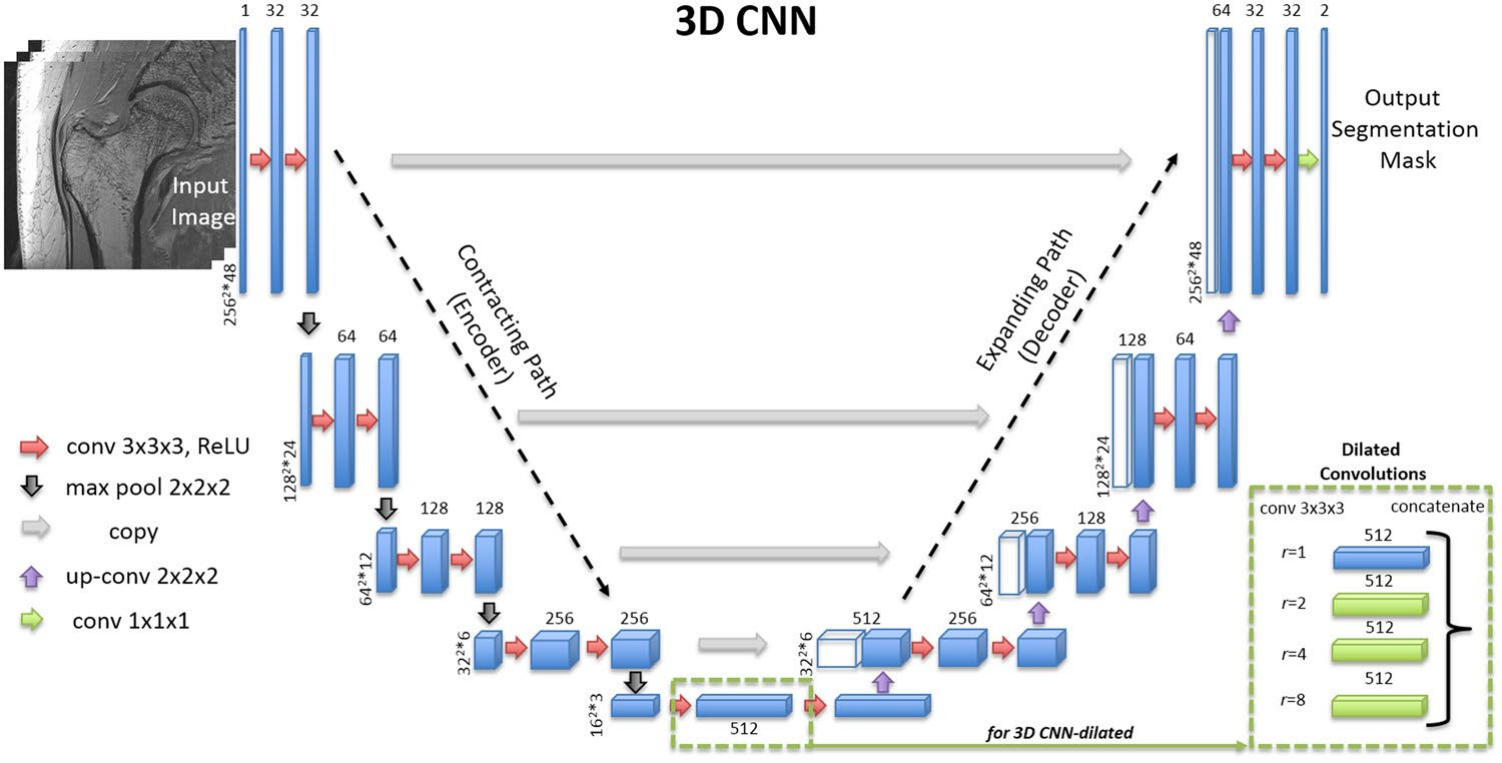
CNN architecture of one of the 3D CNNs used in the paper. Blue rectangles represent feature maps with the size and the number of feature maps indicated. Different operations in the network are depicted by color-coded arrows. The architecture represented here contains 32 feature maps in the first and last layer of the network and 4 layers in the contracting/expanding paths. In 3D CNN-dilated, dilated convolutions with multiple dilation rates are performed and concatenated (as indicated by green dashed boxes) at the center layer of the original 3D CNN.

The first approach (2D CNN) uses a so-called U-net architecture^38^ which was built upon a fully convolutional network (FCN)^63^. In the U-net architecture, the network uses a set of larger images as input and starts with a contracting path (encoder) similar to the conventional pyramidal CNN architectures^64^. Each pooling operation is followed by two convolutional layers with twice as many feature maps. After the contracting path, the network starts to expand in a way more or less symmetric to the contracting path (decoder), with some cropping and copying from the contracting path. The output of the 2D CNN is a trabecular bone probability map of the center area of the input image. The size of the center area depends on the number of layers in the contracting/expanding paths. In addition, we experimented the use of padded convolutions as opposed to the unpadded ones in 2D CNN. The use of padded convolutions provide the trabecular bone probability map of the whole 2D image as an output. The second approach (3D CNN), illustrated in Fig. 6, is the extension of 2D CNN into three dimensions for volumetric segmentation using three-dimensional convolution, up-convolution and max-pooling layers^42^. In the 3D CNN, we use padded convolutions as opposed to unpadded ones proposed in^42^ in order to provide a trabecular bone probability map of the whole image as an output. The third approach (2D/3D CNN-dilated) extends the 2D/3D CNN with the addition of dilated^43^ (also known as atrous)^44^ convolutions at the center layer of the architecture where the encoder and decoder network meets. Dilated convolutions are used to enlarge the receptive field of the convolutions to provide superior global connectivity and multi-scale context information of the input image during architecture design^65,66^. Incorporating dilated convolutions on the center layer where the representation of the input is highly dense due to encoding is expected to provide multi-scale global proximal femur segmentation information by expanding the receptive field effectively.

In all the CNNs, we use horizontal flipping for data augmentation^67^ since our dataset contained images from subjects who had been scanned either at the right hip or left hip. The initialization of the convolution kernel weights is known to be important to achieve convergence. In all experiments, we use the so-called Xavier^68^ weight initialization method. The Xavier initializer is designed to keep the scale of the gradients roughly the same in all layers. This prevents the vanishing gradient^69^, enabling effective learning. As proposed in the original U-net article^38^, in the 2D CNN, we use unpadded 3 × 3 convolutions and 2 × 2 max-pooling operations with stride 2 to gradually decrease the size of the feature maps. In the expanding path, upsampling the feature map size is followed by an unpadded 2 × 2 up-convolution that halves the number of feature maps. For the 3D CNN, padded 3 × 3 × 3 convolutions and up-convolutions, 2 × 2 × 2 max-pooling with stride 2 are used in contrast to unpadded operations as proposed in^42^ and^38^. Padded operations enable the size of the output trabecular bone mask to be equal to the input image size. This removes the requirement of using mirrored images during inference. For the 3D CNN-dilated, a variant of spatial pyramid pooling^70^ was used to replace the center layer of the 3D CNN architecture (Fig. 6). Multiple padded 3 × 3 × 3 convolutions with the number of feature maps equal to the original 3D CNN implementation were concatenated using dilation rates, *r* = 1, 2, 4, 8. Experiments were performed to analyze the effect of combining different dilation rates and the number of layers in the encoder-decoder architecture. For non-linearly transforming data within each layer of the CNN, rectifier linear unit (ReLU)^71^ is used as an activation function. ReLU is defined as $f(x)={\rm{\max }}\,\mathrm{(0,}\,x)$. In the last layer of the CNN, we use softmax to compute the conditional distribution over the voxel label.

The output of the softmax layer from the CNN is used to define a loss function which aims to minimize the error between the ground truth and the automatic segmentation via training. In our implementation, a loss function is defined as a negative log-probability of a target label (ground-truth) from an expert manually-segmented MR image. In medical images, the anatomical structure of interest usually occupies a small portion of the image. This potentially biases the CNN prediction towards background which constitutes the large portion of the images. To overcome this imbalanced class problem, we re-weighted the loss function during training. We achieve this by incorporating the number of proximal femur, *N*_*p*_, and background, *N*_*b*_, voxels into the loss value such that the error in voxels belonging to the trabecular bone are given more importance:

1 $$CE=-\,\frac{1}{N}\sum _{i\mathrm{=1}}^{N}\,(\frac{{N}_{b}}{N}{y}_{i}\,\mathrm{log}\,{p}_{i}+\frac{{N}_{p}}{N}\mathrm{(1}-{y}_{i})\,\mathrm{log}\,\mathrm{(1}-{p}_{i}))$$

where *N* is the number of voxels, *y*_*i*_ is a binary variable indicating if the trabecular bone is a correct prediction, *p*_*i*_ is the probability of model prediction to be trabecular bone.

**Figure 7:**
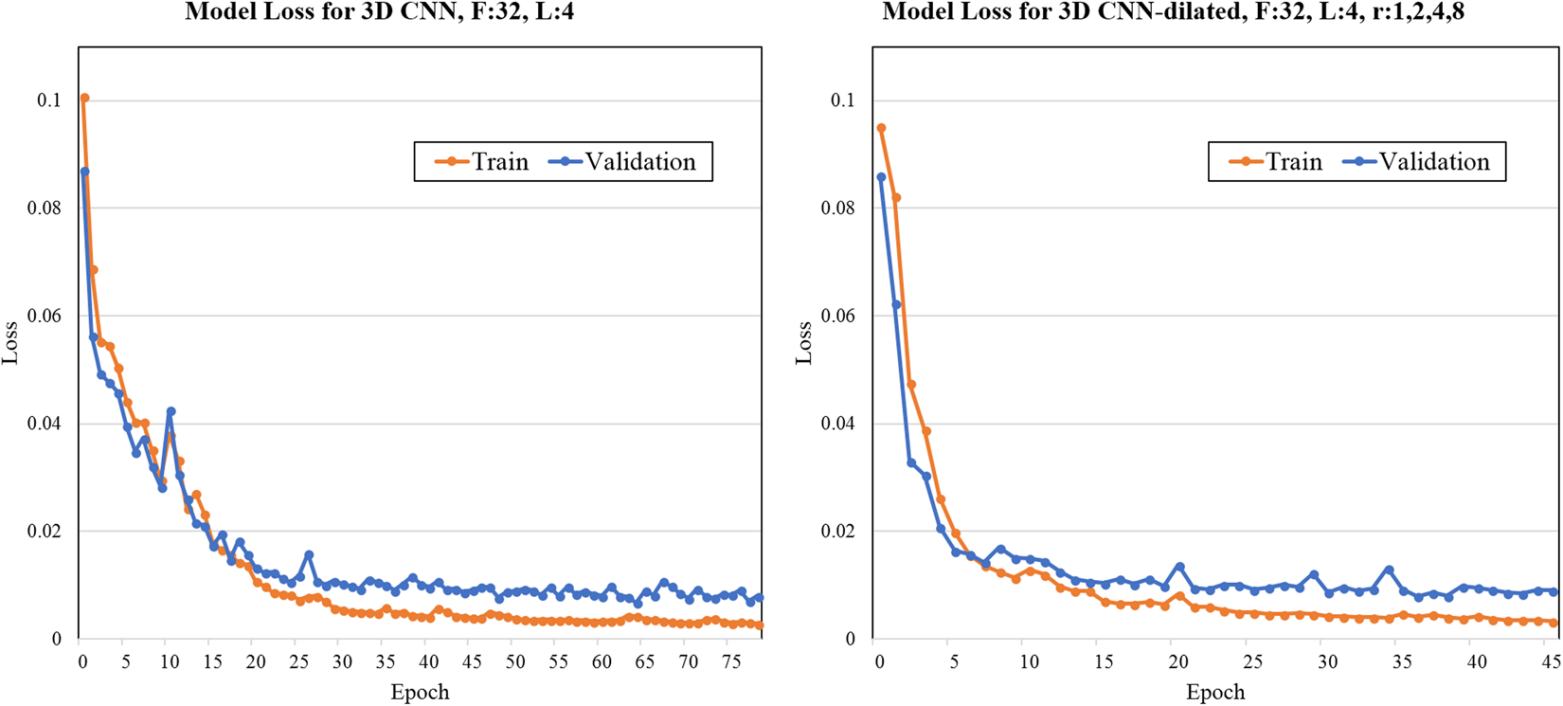
Examples of train and validation loss plots for two different CNNs. Weighted cross-entropy loss was minimized using the Adam algorithm. As indicated by the x-axis, the number of epochs used for training different CNNs differs due to early stopping criteria used during cross-validation. In both cases, as expected, the loss in train dataset is lower that the validation set when the training is stopped.

We use the Tensorflow^53^ software library to implement CNNs. In the minimization of the loss function, we use adaptive moment estimation^72^ (Adam). Parameters used in training the CNNs are outlined in Table 5. Figure 7 provides an example of training and validation loss plots for two different CNN models. We perform experiments on a server using an NVIDIA 16GB Tesla P100 GPU card. For the 2D CNN, we used three consecutive slices and the segmentation mask from the center slice in order to capture some 3D connectivity information from 2D network architecture.

**Table 5:** Hyperparameters used for CNN training.

Phase	Parameter	Value
Initialization	Weights	Xavier
	Bias	0.10
Training	Input Image Size - 2D CNN	512 × 512 × 3
	Input Image Size - 3D CNN	512 × 512 × 48
	Optimizer	Adam
	Batch Size	1
	Learning Rate	5e-5

### Inference and Post-processing

To predict the segmentation of the voxels in the border region of the images, we extrapolate the missing content by mirroring the input image during inference in experiments with the 2D CNN with unpadded convolutions. The probability of any voxel being trabecular bone can be calculated using multiple batches which covers that voxel at the center area of the patch. Because of this reason, during inference we use multiple patches for each voxel and average the probability of that voxel to calculate the probability of that voxel being trabecular bone. In total, we divide the mirrored image into 9 patches that cover the full mirrored image with an ordered overlap. For the padded 2D CNNs and 3D CNNs, mirroring of the images was not required due to the selection of padded convolutions in the network architecture.

We perform basic post-processing on the segmentation results from the 2D CNNs to remove small clusters of misclassified bone regions as indicated by Fig. 4b,c. Since trabecular bone forms a 3D connected volume and covers the most number of voxels at the output of CNN, volumetric constraints are imposed by removing clusters with volumes smaller than the maximum volume of connected labels. The label corresponding to the maximum connected volume within 3D segmentation mask represents the proximal femur. This approach successfully removes those small clusters which were misclassified as proximal femur during the inference. Since using 3D convolution is capable of capturing 3D connectivity information of the trabecular bone accurately, this post-processing step was not required for the experiments based on the 3D CNNs.

### Dataset

This study had institutional review board approval from New York University School of Medicine, and written informed consent was obtained from all subjects. The study was performed in accordance with all regulatory and ethical guidelines for the protection of human subjects by the National Institutes of Health. Images were obtained using commercial 3T MR scanner (Skyra, Siemens, Erlangen) with a 26-element radiofrequency coil setup (18-element Siemens commercial flexible array and 8-elements from the Siemens commercial spine array). High resolution proximal femur microarchitecture T1-weighted 3D fast low angle shot (3D FLASH) images were acquired with the following parameters: TR/TE = 31/4.92 ms; flip angle, 25°; in-plane voxel size, 0.234 mm × 0.234 mm; section thickness, 1.5 mm; matrix size, 512 × 512; number of coronal sections, 60; acquisition time, 25 minutes 30 seconds; bandwidth, 200 Hz/pixel. High resolution acquisitions are required for resolving bone microarchitecture that is fundamental for accurate osteoporosis characterization. Using this imaging protocol, 86 post-menopausal women were scanned. This dataset contains 36 postmenopausal women with clinical osteoporosis. Osteoporosis is defined as the presence of a fragility fracture that was radiographically confirmed (low-energy fracture due to a fall from a standing height). The sites of the fractures were the spine (n = 4), upper extremity (n = 15), lower extremity (n = 12), pelvis/sacrum (n = 1), and ribs (n = 4). The dataset contains either the left or right hip image from each subject. In cases where the subject has fragility fractures on one hip, MR data was acquired on the other hip, where no fragility fractures occurred.

Segmentation of the proximal femur was achieved by manual selection of the trabecular border of bone on MR images by an expert under the guidance of a musculoskeletal radiologist^15^. This resulted in two regions defined as trabecular bone of the proximal femur and the background. The central 48 coronal slices (covering 7.2 cm) were used for segmentation tasks covering the proximal femur and reducing the size of the input image especially for the 3D CNN. Due to memory limitations of the GPU card, we resampled each slice of the MR images into 256 × 256 using bicubic spline interpolation, and used 16 and 32 initial feature maps for the 3D CNN. Analysis of the segmentation results were performed against the original (512 × 512) hand-segmented proximal femur masks.

### Model selection

Four-fold cross-validation is performed to assess the performance of different CNN architectures. Stratified random sampling is used to partition the sample into four disjoint groups. The first two groups have 21 subjects each, and the other two groups have 22 patients each. Each of the four groups serves as a validation set to assess the accuracy of a prediction model obtained from the other three groups combined as a training set. In this way, four separate segmentation models are derived, with each model is applied to segment the proximal femur in a validation set - data independent of the ones that is used to derive the model.

While training the CNNs, we use early stopping in order to prevent over-fitting and to enable fair comparison between different CNN architectures. Training is stopped when the accuracy on the validation set does not improve by 10^−4^ within the last 10 epochs. First 30 epochs are trained without early stopping.

### Evaluation

Manual segmentations of the proximal femur were used as the ground truth to evaluate different CNN structures. We define voxels within the proximal femur and background voxels as positive and negative outcomes, respectively. The performance of CNNs are evaluated using ROC and PRC analysis, DSC, sensitivity/recall, precision and surface-based distance measurements, such as ASD and MSD. The DSC metric^73^, also known as F1-score, measures the similarity/overlap between manual and automatic segmentations. DSC metric is the most widely used metric when validating medical volume segmentations^74^, and it is defined as:

2 $$DSC=2TP/(FP+2TP+FN)$$

where TP, FP, and FN are detected number of true positives, false positives and false negatives, respectively. Sensitivity/recall measures the portion of proximal femur bone voxels in the ground truth that are also identified as a proximal femur bone voxel by the automatic segmentation. Sensitivity/recall is defined as:

3 $$sensitivity/recall=TP/(TP+FN)$$

Similarly, specificity measures the portion of background voxels in the ground truth that are also identified as a background voxel by the automatic segmentation. Specificity is defined as:

4 $$specificity=TN/(TN+FP)$$

Lastly, precision, also known as positive predictive value (PPV), measures the proportion of trabecular bone voxels in the ground truth and voxels identified as trabecular bone by the automatic segmentation. It is defined as:

5 $$precision(PPV)=TP/(TP+FP)$$

ASD provides a measure to identify how much the segmentation surface, *S*, varies from the ground truth surface, *G*, on average. By defining the shortest Euclidean distance of an arbitrary voxel *v* to a surface *S* by $d(v,\,S)=\mathop{{\rm{\min }}}\limits_{s\varepsilon S}\Vert v-s\Vert$, ASD can be written as:

6 $$ASD=\frac{1}{{N}_{S}+{N}_{G}}(\sum _{{x}_{S}\varepsilon S}\,d({x}_{S},\,G)+\sum _{{x}_{G}\varepsilon G}\,d({x}_{G},\,S))$$

where *N*_*S*_ and *N*_*G*_ are the number of segmentation and ground truth surface voxels, respectively. Similarly, MSD also known as the symmetric Hausdorff distance is defined by taking the maximum distance instead of average:

7 $$MSD=\,{\rm{\max }}\{\mathop{{\rm{\max }}}\limits_{{x}_{S}\varepsilon S}\,d({x}_{S},\,G),\mathop{{\rm{\max }}}\limits_{{x}_{G}\varepsilon G}\,d({x}_{G},\,S)\}$$

## Data Availability

The datasets generated during and/or analyzed during the current study are available from the corresponding author on reasonable request.
